# Objectively measured physical activity, sedentary time and subclinical vascular disease: Cross-sectional study in older British men

**DOI:** 10.1016/j.ypmed.2016.05.031

**Published:** 2016-08

**Authors:** Tessa J. Parsons, Claudio Sartini, Elizabeth A. Ellins, Julian P.J. Halcox, Kirsten E. Smith, Sarah Ash, Lucy T. Lennon, S. Goya Wannamethee, I-Min Lee, Peter H. Whincup, Barbara J. Jefferis

**Affiliations:** a UCL Department of Primary Care and Population Health, UCL Medical School, Rowland Hill Street, London NW3 2PF, UK; b UCL Physical Activity Research Group, University College London, London, UK; c Institute of Life Sciences, Swansea University, Singleton Park, Swansea SA2 8PP, UK; d Brigham and Women's Hospital, Harvard Medical School, Boston, USA; e Population Health Research Institute, St George's University of London, Cranmer Terrace, London SW17 0RE, UK

**Keywords:** Physical activity, Sedentary behaviour, Accelerometer, Cardiovascular disease, Epidemiology

## Abstract

Low physical activity (PA) and high levels of sedentary time (ST) are associated with higher cardiovascular disease (CVD) risk among older people. However, their independent contribution and importance of duration of PA and ST bouts remain unclear. We investigated associations between objectively measured PA, ST and non-invasive vascular measures, markers of CVD risk.

Cross-sectional study of 1216 men from the British Regional Heart Study, mean age 78.5 years, measured in 2010–2012. Carotid intima thickness (CIMT), distensibility coefficient (DC) and plaque presence were measured using ultrasound; pulse wave velocity (cfPWV) and augmentation index (AIx) using a Vicorder. PA and ST were measured using hip-worn ActiGraph GT3X accelerometers.

After adjusting for covariates, each additional 1000 steps per day was associated with a 0.038 m/s lower cfPWV (95% CI = − 0.076, 0.0003), 0.095 10^− 3^ kPa^− 1^ higher DC (95% CI = 0.006, 0.185), 0.26% lower AIx (95% CI = − 0.40, − 0.12) and a 0.005 mm lower CIMT (95% CI = − 0.008, − 0.001). Moderate and vigorous PA (MVPA) was associated with lower AIx and CIMT, light PA (LPA) with lower cfPWV and CIMT and ST with higher cfPWV, AIx and CIMT and lower DC. LPA and ST were highly correlated (*r* = − 0.62). The independence of MVPA and ST or MVPA and LPA was inconsistent across vascular measures. Bout lengths for both PA and ST were not associated with vascular measures.

In our cross-sectional study of older men, all PA regardless of intensity or bout duration was beneficially associated with vascular measures, as was lower ST. LPA was particularly relevant for cfPWV and CIMT.

## Highlights

• We examined physical activity, sedentary time and vascular measures in older men.
• Higher PA and lower ST were beneficially associated with cfPWV, DC, AIx and CIMT.
• More minutes of moderate and vigorous PA were associated with a lower AIx and CIMT.
• More light PA was associated with a lower cfPWV and CIMT.
• Bout lengths of MVPA, LPA and ST were not associated with vascular measures.

## 1 Introduction

The role of higher physical activity levels (PA) and lower levels of sedentary time (ST) in reducing the risk of cardiovascular disease (CVD) in middle age is well established (Shiroma and Lee, 2010). Currently, UK physical activity guidelines recommend that older adults accumulate 150 min per week of at least moderate intensity activity in bouts lasting 10 min or more and minimise long periods of sitting (Department of Health, 2011). However, the importance of duration of spells of activity remains uncertain; studies in older adults have not used objectively measured PA to investigate whether bouts lasting 10 min or longer are more beneficial for CVD risk factors than either shorter bouts or total activity. Likewise, there is little information to suggest how often sedentary time should be interrupted, by what level of activity and for how long. In addition, little is known about the independence of PA and ST among older people, who not only have high risk of CVD but also have the highest levels of ST and very low levels of PA with poor adherence to physical activity guidelines (Jefferis et al., 2014). Furthermore, although the evidence base was too sparse to make recommendations about light activity in previous guidelines (Physical Activity Guidelines Advisory Committee, 2008), it may be that light intensity activity also has health benefits in this age-group (de Souto Barreto, 2015).

Non-invasive vascular markers provide valuable proxy indicators of CVD risk, permitting investigation of risk burden and development of subclinical CVD prior to onset of a CVD event. Greater arterial stiffness, measured by carotid femoral pulse wave velocity (cfPWV) (van Sloten et al., 2014), and carotid distensibility coefficient (DC) (van Sloten et al., 2014), wave reflection as indicated by Augmentation Index (AIx) (Wang et al., 2010), increased arterial wall thickening assessed by carotid intima-media thickness (CIMT) (van den Oord et al., 2013), and presence of carotid plaque (Inaba et al., 2012) are all associated with onset of CVD events. However, to date, very few studies have investigated objectively measured PA in relation to non-invasive vascular measures; the few that have are in young or middle-aged adults (Gomez-Marcos et al., 2014, Huynh et al., 2015, Kozakova et al., 2010, Andersson et al., 2015), smaller samples (Gomez-Marcos et al., 2014, Kozakova et al., 2010) or have evaluated a much narrower range of outcome measures (Kozakova et al., 2010, Laursen et al., 2015).

We therefore investigated associations between objectively measured physical activity of different intensities, sedentary time and a range of vascular measures, including cfPWV, DC, AIx, CIMT and presence of plaque, using a large sample of community-dwelling older men. We investigated whether (i) the intensity of activity was related to vascular measures, hypothesising adverse associations for sedentary time, and increasing benefits from light intensity activity upwards and (ii) whether time accumulated in bouts of PA or ST was related to vascular measures in a dose-dependent manner, hypothesising greater benefits from longer bouts of PA or increased risks from longer bouts of ST.

## 2 Material and methods

### 2.1 Sample

The British Regional Heart Study is a prospective, population-based cohort study following up 7735 men (> 99% Caucasian) recruited from primary care practices in 24 British towns in 1978–80. In 2010–2012, 3137 surviving men were invited to a physical examination including non-invasive vascular measurements and to wear a physical activity monitor (accelerometer). A total of 1528 individuals accepted and returned an accelerometer with ≥ 3 days of data; 254 men with pre-existing heart attack, heart failure or stroke were excluded. Of the remaining 1274 men, 1213 had data for all other relevant covariates, leaving 1118–1206 men for data analysis, depending on the vascular outcome. The National Research Ethics Service (NRES) Committee London provided ethical approval. Participants provided informed written consent to the investigation, in accordance with the Declaration of Helsinki.

### 2.2 Non-invasive vascular measures

Left and right carotid arteries were imaged using a Z.One Ultra ultrasound system (Zonare Medical Systems, Mountain View, CA) with a 5- to 10-mHz linear probe. A cross-sectional sweep from the base of the common carotid artery to the jaw bone and longitudinal images of the common carotid artery approximately 1 cm proximal to the carotid bifurcation were recorded. Ipsilateral brachial blood pressures were taken immediately after each carotid assessment (Omron HEM 907 recorder, mmHg). Peak systolic and end-diastolic common carotid artery diameter and CIMT were measured using Carotid Analyser software (Medical Imaging Applications, Iowa City, IA). From the longitudinal images, a region of interest (5–10 mm) was selected in a plaque free area, at least 1 cm from the bifurcation. CIMT was measured in three end-diastolic images on each side and the mean calculated. DC was calculated as previously described (Dijk, 2005). Mean values of right and left CIMT and DC were used in analyses. Ultrasound images were reviewed offline for presence of plaque, defined as a focal area of intima medial thickening ≥ 1.2 mm at its thickest point or with ≥ 50% thickness than the adjacent intima medial thickness.

Carotid femoral pulse wave velocity (cfPWV) was assessed using a Vicorder (Skidmore Medical, Bristol UK), with participants semi-supine. A 2 × 9-cm cuff was positioned around the neck with the bladder over the right carotid pulse, and a Hokanson SC10 cuff around the right thigh. Path length was measured from the sternal notch to the centre of the thigh cuff. The cuffs were simultaneously inflated and traces with a minimum of 3 good quality waveforms recorded. Two cfPWV measurements, within ≤ 0.5 m/s of each other, were accepted and averaged.

AIx was measured with participants seated using the Vicorder. A Hokanson SC10 cuff was positioned mid upper right arm, inflated to diastolic pressure, and once good quality waveforms were acquired, the signal was saved. Two recordings with both readings of augmentation pressure and AIx within ≤ 5% of each other were accepted and averaged.

All measures were made by 2 vascular technicians. The coefficients of variation for cfPWV, DC, AIx and CIMT were 4.7%, 12.7%, 14.6% and 7.7%, respectively; the agreement coefficient for presence of plaque was 0.8.

### 2.3 Physical activity

Men wore the GT3X accelerometer (ActiGraph, Pensacola, Florida) over the right hip for 7 days, during waking hours, removing it for swimming or bathing. Accelerometers were set to record movements on the vertical axis every 5 s, and data were integrated into 60 s epochs. Non-wear time was identified as reported previously (Jefferis et al., 2014) and excluded using the R package “Physical Activity” (Choi et al., 2011). Non-wear time was defined as periods of continuous zeros lasting more than 90 min; within these periods, up to 2 min of non-zero counts were allowed as non-wear time if no activity counts were detected during both the 30 min before and after that interval, to allow for the possibility of artefactual monitor movements (e.g., accidental movement of the monitor while left on a table). Therefore, any non-zero counts except the ≤ 2 min allowed within a period of zeros were considered as wear time. Valid wear days were defined as ≥ 600 min wear time, and participants with ≥ 3 valid days (92% of men who received an accelerometer) were included in analyses. Each minute of activity was categorised using intensity threshold values of counts per minute developed for older adults: < 100 for sedentary time (ST) (< 1.5 MET), 100–1040 for light activity (LPA) (1.5–3 MET) and > 1040 for moderate and vigorous activity (MVPA) (≥ 3 MET) (Copeland and Esliger, 2009).

### 2.4 Other measures

Body mass index (BMI, kg/m^2^) was calculated from height (Harpenden stadiometer) and weight in light indoor clothing (Tanita body composition analyser (BC-418 or Tanita scales if the participant had a pacemaker or defibrillator). The average of two seated blood pressure readings (Omron HEM-907 recorder, mmHg) were used. Heart rate (HR) was measured by electrocardiogram. Men self-completed a questionnaire including information about: current cigarette smoking, alcohol consumption, living alone, ever receiving a doctor diagnosis of heart attack, heart failure or stroke (with symptoms lasting > 24 h), narrowing or hardening of the leg arteries (including claudication) (peripheral arterial disease), diabetes and current use of anti-hypertensive medication. Social class was based on longest held occupation at study entry (1978–80) and categorised as manual and non-manual. Region of residence (1978–80) was grouped into Scotland, North, Midlands and South of England.

## 3 Statistical methods

Men reporting a clinical diagnosis of heart attack, heart failure or stroke (with symptoms lasting > 24 h) were excluded from analyses. Descriptive statistics for demographic characteristics, vascular measures, PA and ST were calculated by quartile of daily minutes of ST and MVPA.

We used regression models to investigate associations between each vascular and PA measure; linear regression models for cfPWV, DC, AIx and CIMT and logistic models for plaque. PA exposures investigated were total activity counts per day, steps per day and minutes per day of ST, LPA and MVPA. The mean difference (or OR) for each outcome was estimated for each 10,000 counts of total activity, 1000 steps, 30 min of ST or LPA and 10 min of MVPA. We examined whether associations between vascular measures, and different activity intensities were independent by mutually adjusting for (i) MVPA and ST and (ii) MVPA and LPA in the same model. ST and LPA were not included in the same model due to collinearity (*r* = − 0.62).

Associations between number of minutes accumulated in bouts of MVPA, LPA or ST and the vascular measures were investigated with the following categories of bout durations: ST lasting 1–15, 16–30, 31–60 and ≥ 61 min; LPA lasting 1–9 and ≥ 10 min; and MVPA lasting 1–9 and ≥ 10 min. MVPA bout durations were based on current guidelines (Department of Health, 2011), and those for ST and LPA were selected according to their distributions. All models were adjusted for average accelerometer wear time (minutes/day), season of accelerometer wear, age, region of residence, systolic blood pressure, social class, living alone, smoking status and alcohol consumption. To investigate potential confounding by anti-hypertensive medication or diabetes or whether any effects were mediated by BMI or HR, we made further adjustments for each of these. Models were also repeated after excluding men with peripheral arterial disease.

## 4 Results

Men spent on average 72%, 23% and 5% of their time in ST, LPA and MVPA, respectively, had a mean of 4938 steps and 164,749 accelerometer counts per day (Table 1). Eighty percent of men had 7 days of accelerometer data, and 96% had ≥ 5 days of data. Men who were more sedentary were older and more likely to live alone, smoke, be taking *anti*-hypertensive medication, have diabetes, have higher BMI and HR and have lower SBP and DBP (Table 1). They spent more of their day in ST and less in LPA and MVPA (Table 1). Relationships with MVPA were in the opposite direction (Supplementary Table S1). Men who agreed to participate were younger and 10 years previously had a lower BMI compared to men who did not participate (Sartini et al., 2015). Swimming, reported by 3% of men (half of whom did a single session in the week), was not incorporated into analysis since it was not measured by accelerometer.

### 4.1 PA, ST and non-invasive vascular measures

Men who were more sedentary had higher AIx and CIMT and lower DC (*p* = 0.04, *p* < 0.0001 and *p* = 0.02 respectively, Table 1). Conversely, men with higher levels of MVPA had a lower cfPWV, AIx and CIMT and higher DC and fewer of them had plaque present (*p* ≤ 0.002, Supplementary Table S1). In regression models adjusted for measurement related factors and confounders, PA was inversely and ST positively associated with cfPWV, AIx and CIMT and vice versa for DC, but associations with carotid plaque were not observed (Table 2). Total daily counts and steps were associated with all outcomes except plaque; higher counts and more steps with a lower cfPWV, AIx and CIMT and with a higher DC (Table 2, models 1 and 2). Total daily counts and steps were highly correlated (*r* = 0.95, *p* < 0.0001). ST and LPA were highly correlated (*r* = − 0.62) and therefore not included in the same model.

### 4.2 Arterial stiffness (cfPWV and DC)

Each extra 30 min of LPA per day was associated with a 0.053 m/s lower cfPWV (95% CI = − 0.103, − 0.002) (Table 2, model 4). Associations with ST were in the opposite direction and of a similar magnitude (Table 2, model 5). When models were mutually adjusted, associations between LPA/ST and cfPWV were weakened, and that between ST and DC was abolished (models 6 and 7).

### 4.3 Wave reflection (AIx)

Each 10 min of MVPA per day was associated with a 0.179% lower AIx (95% CI = − 0.297, − 0.061), (Table 2 model 3). Each 10 min of ST was associated with a 0.181% higher AIx (95% CI = 0.038, 0.323) (model 4). When ST and MVPA were mutually adjusted, the association with MVPA was weakened and that with ST abolished (model 6). When LPA and MVPA were included, the association with MVPA persisted (model 7).

### 4.4 CIMT

Each extra 10 min of MVPA per day was associated with a 0.0031 mm lower CIMT (95% CI = − 0.0061, − 0.0001) (Table 2 model 3). An extra 30 min of LPA was associated with a 0.0075 mm lower CIMT (95% CI = − 0.0122, − 0.0029) (model 4); LPA coefficients were slightly smaller than MVPA (when LPA coefficients were divided by 3, to also relate to 10 min increments). The association with ST was in the opposite direction and of similar size to that with LPA (model 5). When LPA or ST and MVPA were mutually adjusted, associations with CIMT persisted for LPA and ST and were abolished for MVPA (models 6 and 7).

### 4.5 General

Additional adjustment for use of anti-hypertensive medication or diabetes status did not change findings. Excluding men with peripheral arterial disease (*n* = 37) made little difference; associations between cfPWV and LPA or ST were slightly weakened and significance borderline. The addition of BMI to the models reduced coefficients for associations between PA/SB and AIx by 30–45% and associations with CIMT by 19–43%, little changed associations with DC and slightly increased coefficients for associations with cfPWV (≤ 17%). Adjustment for HR little changed associations with CIMT strengthened associations with AIx and abolished associations with cfPWV and DC. MVPA minutes were right skewed, so analyses were repeated using square root transformed MVPA, which normalised the distribution. Results were unchanged except that a weak positive association between MVPA and DC became apparent (*p* = 0.04) and the inverse association between MVPA and AIx remained significant after adjusting for ST (*p* = 0.04).

Few men accumulated many bouts of MVPA of ≥ 10 min; 31% accumulated ≥ 5 bouts per week, 12% accumulated ≥ 10 bouts per week. Regression models examining associations between number of minutes accumulated in bouts of MVPA, LPA or ST of particular lengths and vascular measures showed no consistent evidence that accumulating activity in bouts of shorter (or longer) lengths was associated with these measures (Supplementary Table S1).

## 5 Discussion

In this study of older community-dwelling men, higher levels of PA and lower levels of ST were associated with lower cfPWV, AIx and CIMT and higher DC. There was no evidence that accumulating PA or ST in bouts of shorter or longer durations was consistently associated with any of the vascular measures we examined, suggesting that all PA was associated with a lower CVD risk regardless of the pattern in which it was accumulated. We found some evidence that associations between vascular measures and PA were independent of ST and vice versa, although this was not entirely consistent across vascular measures: the associations between MVPA and AIx, ST and CIMT and LPA and CIMT were independent of other PA and ST variables included in the model. LPA and ST were highly inversely correlated and therefore their influence was inter-dependent. LPA in older adults may well require moderate effort and greater energy expenditure than in younger adults. We used cutoff values for LPA and MVPA specifically developed for older adults; (Copeland and Esliger, 2009), which are significantly lower than those frequently used for young or middle-aged adults, but MVPA and LPA still appear relevant to development of arterial disease in men of this age.

The absolute values for cfPWV and CIMT depend on the measurement technique and device used as well as the levels of cardiovascular risk factors in the study sample, although values for our study were in line with other studies: mean cfPWV for men in our study was only a little lower than European reference values for men over 70 years (10.2 vs. 10.9 m/s) (The Reference Values for Arterial Stiffness' Collaboration) and CIMT a little higher than worldwide reference values for a healthy sub-population of men age 80 years (0.81 vs. 0.74 mm) (Engelen et al., 2013).

Of our measures of arterial stiffness/wave reflections, we observed associations between both PA and ST for cfPWV, DC and AIx. Gomez-Marcos et al. found similar associations in middle-aged adults (55 years) between moderate or light activity and AIx, and a detrimental association between ST and AIx, but it was not reported whether these associations were independent of each other (Gomez-Marcos et al., 2014). In line with our findings, some observational studies have reported associations between PA and a lower cfPWV (Andersson et al., 2015, Laursen et al., 2015), although null or weak associations have been reported in smaller studies (Gomez-Marcos et al., 2014). In studies of young and middle-aged adults, vigorous rather than light or moderate PA is reported to be related to arterial stiffness (Huynh et al., 2015, Andersson et al., 2015, Schmitz et al., 2001). In our population, men spent less than 5% of each day in moderate or vigorous activity combined; the low prevalence might explain why we did not find associations between MVPA and cfPWV or DC. Andersson et al. investigated bouts of MVPA in middle-aged adults (mean age 47 years), and while higher MVPA was associated with lower cfPWV, there was no difference in the associations for short (< 10 min) versus long (≥ 10 min) bouts (Andersson et al., 2015), suggesting as in our study, that bout duration may not be relevant to accumulation of physical activity in relation to these vascular measures. We found associations between PA or ST and cfPWV and DC were abolished by adjusting for HR, suggesting these associations are mediated via HR.

Our finding that higher total PA, MVPA and LPA and lower ST levels were associated with lower CIMT are consistent with conclusions of a literature review in which most cross-sectional studies showed a beneficial association between CIMT and self-reported physical activity (Kadoglou et al., 2008). More recent studies of middle-aged adults report varying results; in one study, an association between accelerometer-measured PA and CIMT did not persist after adjusting for potential confounding factors (Gomez-Marcos et al., 2014), while in another study, self-reported PA and CIMT remained associated after adjustment, although for a more limited range of factors (Khalil et al., 2013). Adjustment for adiposity might account for some differences; our findings suggest that some of the association between PA/ST and CIMT may be mediated through BMI. In a longitudinal cross-European countries study, the proportion of time spent in accelerometer-measured ST was associated with a greater CIMT at baseline, while vigorous activity was associated with a lower CIMT progression over 3 years follow-up (Kozakova et al., 2010). In our study population, LPA and ST were highly correlated, so that less ST or more LPA (or vice versa) showed similar associations with CIMT. We found no associations between any activity variable and presence of plaque, possibly because the vast majority of men in our study (87%) had evidence of carotid plaque.

### 5.1 Study limitations and strengths

Our study has the advantage of being based on a large sample of community-dwelling men rather than a clinical group at high risk of CVD or with a specific health condition, but our findings may not be generalisable to younger age-groups or women. Men who were invited to the current follow-up but did not participate, were slightly older and had a slightly higher BMI at a previous follow-up 10 years earlier than men who did participate in the current study, but this is unlikely to change the conclusions of this study. The 7-day accelerometer wear protocol in our study was well adhered to; 96% of men provided the ≥ 5 days of data needed to predict habitual PA/ST. (Hart et al., 2011) We used definitions of PA intensity specifically for older adults (Copeland and Esliger, 2009) since using the higher cut points developed for middle-aged adults results in extremely low levels of MVPA (Jefferis et al., 2014) and may not be appropriate for older age-groups. Previously in this cohort, we have found that similar variables (chronic conditions, mobility limitations, mental health and wellbeing and various activity behaviours) predicted MVPA defined by two different cut points (Jefferis et al., 2014). Because the ActiGraph accelerometer is not designed to differentiate sitting from standing (although it compares well with the Activpal monitor that is; Healy et al., 2011), we verified amounts of ST by changing the definition to < 50 cpm; this changed ST very little, suggesting that in men aged 70–90 years ST was very sedentary and did not include much standing time (Parsons et al., 2016). We were able to adjust for a range of potential confounding factors although the cross-sectional nature of our data limits our ability to determine causality, i.e. whether increased PA improves vascular status or a better vascular status allows more physical activity. The relationships between objectively measured PA or ST and non-invasive vascular measures of CVD risk are under-explored and we were able to go beyond other studies and investigate these relationships in terms of activity of different intensities and bout length by examining how many minutes per day were accumulated in bouts of ST, LPA and MVPA of specific durations.

## 6 Conclusions

Our study suggests that higher levels of PA and lower levels of ST are associated with lower CVD risk, as indicated by non-invasive markers of arterial stiffness and atherosclerosis. We found no evidence that bouts of MVPA lasting ≥ 10 min were important over and above total amounts, or that breaking up sedentary time into shorter bouts was beneficial to the range of vascular measures studied, although we acknowledge that power may be limited. Our findings indicate that all activity matters and that even LPA makes an important contribution to total activity in older men.

## Funding

This work was supported by the British Heart Foundation [PG/13/86/30,546 and RG/13/16/30,528] and the National Institute of Health Research [Post-Doctoral Fellowship 2010–03–023]. IML was partly supported by National Institutes of Health [CA154647]. The funders had no role in the design and conduct of the study; collection, management, analysis and interpretation of the data; preparation, review and approval of or decision to publish the manuscript. The views expressed in this publication are those of the author(s) and not necessarily those of the Funders.

## Disclosures

The authors declare there is no conflict of interest.

## Transparency document

Transparency document. Image 1

## Figures and Tables

**Table 1:** Characteristics of 1274 British men without pre-existing CVD or heart failure, by quartile of minutes per day spent in SB, measured in 2010–2012.

	Quartile of sedentary (minutes/day)						
1	2	3	4
Mean (SD) or % (*n*)	295–<564	≥ 564–<618	≥ 618–<674	≥ 674	*P* (trend)	All men	*N*
*N*^a^	326^a^	321^a^	316^a^	311^a^			1274
Age (years)	77.2 (3.9)	77.7 (4.2)	79.0 (4.6)	79.8 (5.1)	< 0.0001	78.4 (4.6)	1274
Manual Social class,% (*n*)	48.3 (157)	42.2 (135)	48.6 (152)	48.7 (150)	0.28^b^	46.9 (594)	1266
Lives alone, % (*n*)	15.6 (50)	15.5 (49)	20.7 (65)	24.2 (74)	0.01^b^	19.0 (238)	1256
Smoker, % (*n*)	1.9 (6)	1.6 (5)	4.5 (14)	6.6 (20)	0.002^c^	3.6 (45)	1257
Alcohol (units per week)	6.5 (8.1)	6.9 (8.1)	6.1 (7.1)	5.9 (7.2)	0.17	6.4 (7.6)	1240
BMI (kg/m^2^)	26.5 (3.2)	27.1 (3.7)	27.3 (3.8)	27.7 (4.4)	< 0.0001	27.1 (3.8)	1263
Heart rate (bpm)	63.3 (11.0)	64.6 (11.0)	64.3 (12)	65.9 (11.9)	0.01	64.5 (11.5)	1115
Systolic blood pressure (mmHg)	149.1 (17.9)	148.1 (18.4)	146.4 (17.8)	145.9 (20.8)	0.02	147 (19)	1271
Diastolic blood pressure (mmHg)	79.2 (11.2)	77.9 (11.2)	76.8 (11.2)	76.3 (12.2)	0.001	77 (11)	1271
Taking anti-hypertensives, % (*n*)	48 (158)	51 (165)	56 (178)	61 (189)	0.01^b^	54.2 (690)	1274
Diabetes, % (*n*)	10.4 (34)	14.0 (45)	13.3 (42)	18.7 (58)	0.03^b^	14.1 (179)	1273
cfPWV (m/s)	10.18 (1.68)	10.09 (1.55)	10.21 (1.74)	10.38 (1.79)	0.10	10.21 (1.69)	1174
DC (10^− 3^ kPa^− 1^)	12.39 (3.97)	12.62 (4.39)	12.25 (4.17)	11.67 (4.26)	0.02	12.24 (4.21)	1251
AIx (%)	20.30 (5.71)	20.72 (6.29)	21.47 (6.62)	21.14 (6.12)	0.04	20.90 (6.20)	1264
CIMT (mm)	0.78 (0.14)	0.80 (0.16)	0.81 (0.17)	0.83 (0.15)	< 0.0001	0.81 (0.16)	1256
Plaque present, % (*n*)	83.2 (257)	86.6 (264)	88.6 (272)	89.1 (261)	0.12^b^	86.8 (1054)	1214
Total activity (counts per minute)	243,509 (106,704)	176,267 (87,380)	135,881 (69,693)	99,634 (62,857)	< 0.0001	164,749 (99,271)	1274
Steps/day	6949 (2906)	5324 (2485)	4224 (2123)	3158 (2036)	< 0.0001	4938 (2794)	1274
% time spent sedentary	62.0 (7.2)	70.6 (5.5)	75.5 (5.7)	81.2 (6.0)	< 0.0001	72.2 (9.3)	1274
% time LPA	30.2 (5.5)	24.5 (4.6)	21.0 (4.7)	16.5 (4.9)	< 0.0001	23.1 (7.0)	1274
% time MVPA	7.8 (4.3)	4.9 (3.0)	3.5 (2.4)	2.3 (2.0)	< 0.0001	4.7 (3.7)	1274
ST (min/day)	511 (45)	591 (16)	645 (16)	723 (43)	< 0.0001	616 (84)	1274
LPA (min/day)	252 (60)	209 (50)	182 (50)	149 (51)	< 0.0001	199 (65)	1274
MVPA (min/day)^d^	66 (38)	42 (28)	31 (23)	21 (19)	< 0.0001	40 (33)	1274

BMI, body mass index.

cfPWV, carotid femoral pulse wave velocity.

DC, carotid distensibility coefficient.

AIx, augmentation Index.

CIMT, carotid intima medial thickness.

ST, sedentary time.

LPA, light physical activity.

MVPA, moderate and vigorous physical activity.

a Maximum *N* in quartile, varies slightly with missing covariate data.

b Pearson chi-square test.

c Fisher's exact test.

**Table 2:** Associations between physical activity intensity, sedentary time and non-invasive vascular measures, *β* (95% CI) from linear regression analyses, OR (95% CI) from logistic regression analyses.

	cfPWV (m/s) *N* = 1118		DC (10^− 3^ kPa^− 1^) *N* = 1193		AIx (%) *N* = 1206		CIMT (mm) *N* = 1197		Plaque *N* = 1156	
*β*	(95% CI)	*β*	(95% CI)	*β*	(95% CI)	*β*	(95% CI)	OR	(95% CI)
**Model 1**										
Total vertical counts (per 10,000/day)	**− 0.012**	**(− 0.023,-0.002)**	**0.031**	**(0.005,0.056)**	**− 0.065**	**(− 0.105,-0.025)**	**− 0.0014**	**(− 0.0024,-0.0004)**	1.00	(0.98,1.01)

**Model 2**										
Steps (per 1000 per day)	− 0.038	(− 0.076,0.000)	**0.095**	**(0.006,0.185)**	**− 0.257**	**(− 0.399,-0.116)**	**− 0.0045**	**(− 0.0081,-0.0010)**	0.99	(0.93,1.06)

**Model 3**										
Total MVPA (/10 min/day)	− 0.022	(− 0.054,0.010)	0.064	(− 0.010,0.138)	**− 0.179**	**(− 0.297,-0.061)**	**− 0.0031**	**(− 0.0061,-0.0001)**	0.99	(0.94,1.05)

**Model 4**										
Total LPA (/30 min/day)	**− 0.053**	**(− 0.103,-0.002)**	0.103	(− 0.013,0.220)	− 0.155	(− 0.339,0.030)	**− 0.0075**	**(− 0.0122,-0.0029)**	0.96	(0.88,1.05)

**Model 5**										
Total ST (/30 min/day)	**0.043**	**(0.004,0.082)**	**− 0.094**	**(− 0.185,-0.003)**	**0.181**	**(0.038,0.323)**	**0.0061**	**(0.0025,0.0097)**	1.03	(0.96,1.10)

**Model 6**										
Total MVPA (/10 min/day)	0.004	(− 0.040,0.048)	0.021	(− 0.082,0.124)	− 0.146	(− 0.310,0.018)	0.0007	(− 0.0034,0.0048)	1.01	(0.94,1.10)
Total ST (/30 min/day)	0.046	(− 0.008,0.101)	− 0.076	(− 0.202,0.050)	0.057	(− 0.142,0.256)	**0.0066**	**(0.0016,0.0116)**	1.04	(0.94,1.15)

**Model 7**										
Total MVPA (/10 min/day)	− 0.011	(− 0.045,0.023)	0.046	(− 0.034,0.126)	**− 0.165**	**(− 0.292,-0.038)**	− 0.0015	(− 0.0047,0.0016)	1.00	(0.94,1.06)
Total LPA (/30 min/day)	− 0.046	(− 0.101,0.008)	0.076	(− 0.050,0.202)	− 0.057	(− 0.256,0.142)	**− 0.0066**	**(− 0.0116,-0.0016)**	0.96	(0.87,1.06)

Men reporting previous diagnosis of heart attack, heart failure and stroke are excluded.

All coefficients adjusted for average daily accelerometer wear time, season of wear, region of residence, age, systolic blood pressure, social class, living alone, tobacco and alcohol consumption.

cfPWV, carotid femoral pulse wave velocity.

DC, carotid distensibility coefficient.

AIx, augmentation index.

CIMT, carotid intima medial thickness.

MVPA, moderate and vigorous physical activity.

LPA, light physical activity.

ST, sedentary time.

## Appendix A Supplementary data

Supplementary tables. Image 2

## References

[bb0005] Andersson C., Lyass A., Larson M.G., Spartano N.L., Vita J.A., Benjamin E.J., Murabito J.M., Esliger D.W., Blease S.J., Hamburg N.M., Mitchell G.F., Vasan R.S. (2015). Physical activity measured by accelerometry and its associations with cardiac structure and vascular function in young and middle-aged adults. J. Am. Heart Assoc..

[bb0010] Choi L., Liu Z., Matthews C.E., Buchowski M.S. (2011). Physical Activity: Process Physical Activity Accelerometer Data (0.1–1).

[bb0015] Copeland J.L., Esliger D.W. (2009). Accelerometer assessment of physical activity in active, healthy older adults. J. Aging Phys. Act..

[bb0020] de Souto Barreto P. (2015). Global health agenda on non-communicable diseases: has who set a smart goal for physical activity?. BMJ.

[bb0025] Department of health (2011). Start Active, Stay Active: A Report on Physical Activity from the Four Home Countries' Chief Medical Officers.

[bb0030] Dijk J.M., Algra A., van der Graaf Y., Grobbee D.E., Bots M.L., SMART study group (2005). Carotid stiffness and the risk of new vascular events in patients with manifest cardiovascular disease. The SMART study. Eur. Heart J..

[bb0035] Engelen L., Ferreira I., Stehouwer C.D., Boutouyrie P., Laurent S. (2013). On behalf of the reference values for arterial measurements collaboration. Reference intervals for common carotid intima-media thickness measured with echotracking: relation with risk factors. Eur. Heart J..

[bb0040] Gomez-Marcos M.A., Recio-Rodriguez J.I., Patino-Alonso M.C., Agudo-Conde C., Lasaosa-Medina L., Rodriguez-Sanchez E., Maderuelo-Fernandez J.A., Garcia-Ortiz L. (2014). Relationship between objectively measured physical activity and vascular structure and function in adults. Atherosclerosis.

[bb0045] Hart T.L., Swartz A.M., Cashin S.E., Strath S.J. (2011). How many days of monitoring predict physical activity and sedentary behaviour in older adults?. Int. J. Behav. Nutr. Phys. Act..

[bb0050] Healy G.N., Clark B.K., Winkler E.A., Gardiner P.A., Brown W.J., Matthews C.E. (2011). Measurement of adults' sedentary time in population-based studies. Am. J. Prev. Med..

[bb0055] Huynh Q.L., Blizzard C.L., Raitakari O., Sharman J.E., Magnussen C.G., Dwyer T., Juonala M., Kahonen M., Venn A.J. (2015). Vigorous physical activity and carotid distensibility in young and mid-aged adults. Hypertens. Res..

[bb0060] Inaba Y., Chen J.A., Bergmann S.R. (2012). Carotid plaque, compared with carotid intima-media thickness, more accurately predicts coronary artery disease events: a meta-analysis. Atherosclerosis.

[bb0065] Jefferis B.J., Sartini C., Lee I.M., Choi M., Amuzu A., Gutierrez C., Casas J.P., Ash S., Lennnon L.T., Wannamethee S.G., Whincup P.H. (2014). Adherence to physical activity guidelines in older adults, using objectively measured physical activity in a population-based study. BMC Public Health.

[bb0070] Kadoglou N.P., Iliadis F., Liapis C.D. (2008). Exercise and carotid atherosclerosis. Eur. J. Vasc. Endovasc. Surg..

[bb0075] Khalil A., Huffman M.D., Prabhakaran D., Osmond C., Fall C.H., Tandon N., Lakshmy R., Prabhakaran P., Biswas S.K., Ramji S., Sachdev H.S., Bhargava S.K., New Delhi Birth Cohort (2013). Predictors of carotid intima-media thickness and carotid plaque in young indian adults: the New Delhi Birth Cohort. Int. J. Cardiol..

[bb0080] Kozakova M., Palombo C., Morizzo C., Nolan J.J., Konrad T., Balkau B., Investigators R. (2010). Effect of sedentary behaviour and vigorous physical activity on segment-specific carotid wall thickness and its progression in a healthy population. Eur. Heart J..

[bb0085] Laursen A.S., Hansen A.L., Wiinberg N., Brage S., Sandbaek A., Lauritzen T., Witte D.R., Jorgensen M.E., Johansen N.B. (2015). Higher physical activity is associated with lower aortic stiffness but not with central blood pressure: the addition-pro study. Medicine (Baltimore).

[bb0090] Parsons T.J., Sartini C., Ellins E.A., Halcox J.P., Smith K.E., Ash S., Lennon L.T., Wannamethee S.G., Lee I.M., Whincup P.H., Jefferis B.J. (2016). Objectively measured physical activity and sedentary behaviour and ankle brachial index: cross-sectional and longitudinal associations in older men. Atherosclerosis.

[bb0095] Physical activity guidelines advisory committee (2008). Physical activity guidelines advisory committee report, 2008. http://www.health.gov/paguidelines/Report/pdf/CommitteeReport.pdf;.

[bb0100] Sartini C., Wannamethee S.G., Iliffe S., Morris R.W., Ash S., Lennon L., Whincup P.H., Jefferis B.J. (2015). Diurnal patterns of objectively measured physical activity and sedentary behaviour in older men. BMC Public Health.

[bb0105] Schmitz K.H., Arnett D.K., Bank A., Liao D., Evans G.W., Evenson K.R., Stevens J., Sorlie P., Folsom A.R. (2001). Arterial distensibility and physical activity in the aric study. Med. Sci. Sports Exerc..

[bb0110] Shiroma E.J., Lee I.M. (2010). Physical activity and cardiovascular health: lessons learned from epidemiological studies across age, gender, and race/ethnicity. Circulation.

[bb0115] The Reference Values for Arterial Stiffness' Collaboration. Determinants of pulse wave velocity in healthy people and in the presence of cardiovascular risk factors: ‘establishing normal and reference values’. Eur. Heart J.;31:2338–2350. 10.1093/eurheartj/ehq165 PMC2948201 20530030

[bb0120] van den Oord S.C., Sijbrands E.J., ten Kate G.L., van Klaveren D., van Domburg R.T., van der Steen A.F., Schinkel A.F. (2013). Carotid intima-media thickness for cardiovascular risk assessment: systematic review and meta-analysis. Atherosclerosis.

[bb0125] van Sloten T.T., Schram M.T., van den Hurk K., Dekker J.M., Nijpels G., Henry R.M., Stehouwer C.D. (2014). Local stiffness of the carotid and femoral artery is associated with incident cardiovascular events and all-cause mortality: the Hoorn study. J. Am. Coll. Cardiol..

[bb0130] Wang K.L., Cheng H.M., Sung S.H., Chuang S.Y., Li C.H., Spurgeon H.A., Ting C.T., Najjar S.S., Lakatta E.G., Yin F.C., Chou P., Chen C.H. (2010). Wave reflection and arterial stiffness in the prediction of 15-year all-cause and cardiovascular mortalities: a community-based study. Hypertension.

