# Supplementary material for: Objectively measured physical activity, sedentary time and subclinical vascular disease: Cross-sectional study in older British men
Source: Prev Med. 2016 Aug;89:194–9. doi: 10.1016/j.ypmed.2016.05.031 (PMC4976831; doi:10.1016/j.ypmed.2016.05.031)
Supplement: Supplementary file 2 — Supplementary tables. [file mmc2.docx]

**ONLINE SUPPLEMENT**

**Table S1. Characteristics of 1274 British men without pre-existing CVD or heart failure, by quartile of minutes per day spent in MVPA, measured in 2010-2012.**

|  | **Quartile of MVPA (minutes/day)** | | | |  |  |
| --- | --- | --- | --- | --- | --- | --- |
|  | **1** | **2** | **3** | **4** |  |  |
| Mean (SD) or % (n) | 0.4 – <3.1^a^ | ≥3.1 - <30.8^a^ | ≥30.8 – <53.5 ^a^ | ≥53.5 ^a^ | **P (trend)** | **N** |
| N* | 291* | 308* | 340* | 335* |  | 1274 |
| Age (years) | 81.0 (5.0) | 78.7 (4.7) | 77.8 (4.0) | 76.5 (3.5) | <0.0001 | 1274 |
| Manual Social class,% (n) | 52 (150) | 45 (139) | 45 (154) | 46 (151) | 0.29^†^ | 1274 |
| Lives alone, % (n) | 23 (65) | 19 (59) | 19 (62) | 16 (52) | 0.18^†^ | 1256 |
| Smoker, % (n) | 6.6 (19)) | 4.6 (14) | 1.5 (5) | 2.1 (7) | 0.002^‡^ | 1257 |
| Alcohol (units per week) | 5.2 (7.3) | 6.0 (7.7) | 6.8 (7.5) | 7.2 (7.9) | <0.0001 | 1240 |
| BMI (kg/m^2^) | 28.2 (4.6) | 27.4 (3.6) | 26.9 (3.6) | 26.1 (3.1) | <0.0001 | 1263 |
| Heart rate (bpm) | 67.1 (13.6) | 65.0 (11.1) | 63.2 (11.0) | 63.1 (10.0) | <0.0001 | 1261 |
| Systolic blood pressure (mmHg) | 143.8 (20.9) | 146.4 (17.8) | 148.7 (18.2) | 150.2 (17.8) | <0.0001 | 1271 |
| Diastolic blood pressure (mmHg) | 74.2 (12.4) | 77.7 (11.1) | 78.5 (11.4) | 79.5 (10.5) | <0.0001 | 1271 |
| Taking anti-hypertensives, % (n) | 66 (192) | 54 (167) | 55 (187) | 43 (144) | <0.0001 | 1274 |
| Diabetes, % (n) | 21.7 (63) | 10.7 (33) | 14.4 (49) | 10.2 (34) | <0.0001 | 1273 |
| cfPWV (m/s) | 10.4 (1.7) | 10.3 (1.8) | 10.3 (1.6) | 10.0 (1.6) | 0.001 | 1174 |
| DC (10^-3^ kPa^-1^) | 11.5 (4.4) | 12.0 (3.9) | 12.7 (4.5) | 12.7 (3.9) | <0.0001 | 1251 |
| AIx (%) | 21.6 (7.0) | 21.2 (5.7) | 20.7 (5.9) | 20.2 (6.1) | 0.002 | 1264 |
| CIMT (mm) | 0.85 (0.17) | 0.80 (0.16) | 0.80 (0.15) | 0.78 (0.14) | <0.0001 | 1256 |
| Plaque present, % (n) | 92.8 (256) | 84.4 (243) | 88.7 (291) | 82.0 (264) | 0.001^†^ | 1214 |
| Total activity (counts per minute) | 61,669 (24,590) | 113,645 (23,416) | 171,554 (29,976) | 294,370 (83,994) | <0.0001 | 1274 |
| Steps/day | 1895 (883) | 3646 (832) | 5302 (1022) | 8401 (2370) | <0.0001 | 1274 |
| % time spent sedentary | 81.8 (6.7) | 75.1 (5.6) | 70.4 (5.7) | 63.0 (7.5) | <0.0001 | 1274 |
| % time LPA | 17.3 (6.5) | 22.2 (5.5) | 24.8 (5.7) | 27.3 (6.5) | <0.0001 | 1274 |
| % time MVPA | 0.8 (0.4) | 2.6 (0.6) | 4.8 (0.8) | 9.7 (3.1) | <0.0001 | 1274 |
| ST (mins/day) | 676 (76) | 638 (65) | 607 (68) | 552 (76) | <0.0001 | 1274 |
| LPA (mins/day) | 144 (56) | 189 (50) | 214 (52) | 239 (61) | <0.0001 | 1274 |
| MVPA (mins/day) | 6.9 (3.7) | 22.3 (4.8) | 41.4 (6.5) | 84.7 (26.9) | <0.0001 | 1274 |

*maximum N in quartile, varies slightly with missing covariate data

^†^Pearson chi square test

^‡^Fisher’s exact test

BMI, body mass index

cfPWV, carotid femoral pulse wave velocity

DC, carotid distensibility coefficient

AIx, augmentation Index

CIMT, carotid intima medial thickness

ST, sedentary time

LPA, light physical activity

MVPA, moderate and vigorous physical activity

**Table S2. Associations between bouts of physical activity, sedentary time, and non-invasive vascular measures.**

|  | **cfPWV (m/s)**  **N=1118** | | **DC** | **(10^-3^ kPa-1)**  **N=1193** | **AIx (%)**  **N=1206** | | **CIMT (mm)**  **N=1197** | | **Plaque**  **N=1158** | |
| --- | --- | --- | --- | --- | --- | --- | --- | --- | --- | --- |
|  | **β** | **(95% CI)** | **β** | **(95% CI)** | **β** | **(95% CI)** | **β** | **(95% CI)** | **OR** | **(95% CI)** |
| **Model 1** |  |  |  |  |  |  |  |  |  |  |
| MVPA mins in bouts 1-9 mins | -0.003 | (-0.008,0.002) | 0.010 | (-0.001,0.021) | -0.012 | (-0.030,0.006) | 0.0002 | (-0.0006,0.0003) | 0.996 | (0.988,1.004) |
| MVPA mins in bouts 10+ mins | -0.001 | (-0.007,0.006) | 0.001 | (-0.015,0.016) | -0.028 | (-0.053,-0.003) | -0.0006 | (-0.0012,0.0001) | 1.005 | (0.993,1.018) |
| Wald test^*^ |  | 0.35 |  | 0.17 |  | 0.008 |  | 0.08 |  | 0.54 |
| Wald test^†^ |  | 0.63 |  | 0.40 |  | 0.37 |  | 0.38 |  | 0.29 |
|  |  |  |  |  |  |  |  |  |  |  |
| **Model 2** |  |  |  |  |  |  |  |  |  |  |
| LPA mins in bouts 1-9 mins | -0.001 | (-0.003,0.002) | 0.007 | (0.001,0.013) | -0.006 | (-0.016,0.003) | -0.0003 | (-0.0005,-0.0000) | 1.000 | (0.995,1.005) |
| LPA mins in bouts 10+ mins | -0.005 | (-0.012,0.001) | -0.008 | (-0.023,0.007) | -0.002 | (-0.026,0.023) | -0.0002 | (-0.0008,0.0004) | 0.995 | (0.984,1.007) |
| Wald test^*^ |  | 0.06 |  | 0.06 |  | 0.25 |  | 0.007 |  | 0.59 |
| Wald test^†^ |  | 0.26 |  | 0.12 |  | 0.76 |  | 0.94 |  | 0.54 |
|  |  |  |  |  |  |  |  |  |  |  |
| **Model 3** |  |  |  |  |  |  |  |  |  |  |
| ST mins in bouts 1-15 mins | 0.001 | (-0.002,0.005) | -0.001 | (-0.008,0.007) | 0.010 | (-0.002,0.022) | 0.0002 | (-0.0000,0.0005) | 1.002 | (0.996,1.007) |
| ST mins in bouts 16-30 mins | 0.000 | (-0.003,0.004) | 0.000 | (-0.007,0.008) | 0.012 | (0.000,0.024) | -0.0001 | (-0.0004,0.0002) | 1.008 | (1.002,1.014) |
| ST mins in bouts 31-60 mins | 0.003 | (0.000,0.005) | -0.005 | (-0.010,0.001) | 0.003 | (-0.006,0.011) | 0.0004 | (0.0002,0.0006) | 1.000 | (0.995,1.004) |
| ST mins in bouts 61+ mins | 0.001 | (-0.001,0.003) | -0.002 | (-0.006,0.002) | 0.008 | (0.002,0.015) | 0.0002 | (-0.0000,0.0003) | 1.001 | (0.997,1.004) |
| Wald test^*^ |  | 0.19 |  | 0.20 |  | 0.07 |  | 0.002 |  | 0.13 |
| Wald test^†^ |  | 0.67 |  | 0.60 |  | 0.47 |  | 0.11 |  | 0.08 |

Men reporting previous diagnosis of heart attack, heart failure, stroke are excluded

All coefficients adjusted for average daily accelerometer wear time, season of wear, region of residence, age, systolic blood pressure, social class, living alone, tobacco and alcohol consumption.

cfPWV, carotid femoral pulse wave velocity

DC, carotid distensibility coefficient

AIx, augmentation Index

CIMT, carotid intima medial thickness

ST, sedentary time

LPA, light physical activity

MVPA, moderate and vigorous physical activity

^*^Wald test for coefficients jointly equal to zero

^†^Wald test for coefficients equal to each other
